# The X-Linked Autism Protein KIAA2022/KIDLIA Regulates Neurite Outgrowth via N-Cadherin and δ-Catenin Signaling

**DOI:** 10.1523/ENEURO.0238-16.2016

**Published:** 2016-10-28

**Authors:** James Gilbert, Heng-Ye Man

**Affiliations:** 1 Department of Biology, Boston University, 5 Cummington Mall, Boston, MA 02215; 2 Department of Pharmacology and Experimental Therapeutics, Boston University School of Medicine, Boston, MA 02118

**Keywords:** autism, dendrite growth, intellectual disability, KIAA2022, KIDLIA, N-cadherin

## Abstract

Our previous work showed that loss of the KIAA2022 gene protein results in intellectual disability with language impairment and autistic behavior (KIDLIA, also referred to as XPN). However, the cellular and molecular alterations resulting from a loss of function of KIDLIA and its role in autism with severe intellectual disability remain unknown. Here, we show that KIDLIA plays a key role in neuron migration and morphogenesis. We found that KIDLIA is distributed exclusively in the nucleus. In the developing rat brain, it is expressed only in the cortical plate and subplate region but not in the intermediate or ventricular zone. Using *in utero* electroporation, we found that short hairpin RNA (shRNA)-mediated knockdown of KIDLIA leads to altered neuron migration and a reduction in dendritic growth and disorganized apical dendrite projections in layer II/III mouse cortical neurons. Consistent with this, in cultured rat neurons, a loss of KIDLIA expression also leads to suppression of dendritic growth and branching. At the molecular level, we found that KIDLIA suppression leads to an increase in cell-surface N-cadherin and an elevated association of N-cadherin with δ-catenin, resulting in depletion of free δ-catenin in the cytosolic compartment. The reduced availability of cytosolic δ-catenin leads to elevated RhoA activity and reduced actin dynamics at the dendritic growth cone. Furthermore, in neurons with KIDLIA knockdown, overexpression of δ-catenin or inhibition of RhoA rescues actin dynamics, dendritic growth, and branching. These findings provide the first evidence on the role of the novel protein KIDLIA in neurodevelopment and autism with severe intellectual disability.

## Significance Statement

Autism spectrum disorder (ASD) is a neurodevelopmental impairment with a strong genetic basis. The cellular and molecular mechanisms linking autism and intellectual disability–related genes to impairments in brain development remain to be fully elucidated. This is the first study to examine the distribution, expression, and neurobiological function of KIAA2022/KIDLIA, a novel gene protein implicated in ASD and intellectual disability. We report that KIDLIA is a neuron-specific nuclear protein expressed in the subplate and cortical plate in the developing brain. Loss of KIDLIA expression impairs neuron migration, dendritic growth, and morphogenesis via regulation of the N-cadherin/δ-catenin signaling pathway and actin dynamics.

## Introduction

Autism spectrum disorder (ASD) is described on the basis of its three core symptoms: diminished language and communication, impaired social interactions, and the tendency for repetitive behaviors (Lord et al., 1989). ASD is becoming increasingly devastating due to its high prevalence, impact on families, and cost to society. Approximately one in 68 individuals in the United States has ASD, and roughly 30% of those with ASD have intellectual disability (ID; Baio, 2014).

The mammalian X chromosome is enriched with genes expressed in the brain, as demonstrated by the high incidence of X-linked ID (Skuse, 2005; Nguyen and Disteche, 2006). Previous work from our group and others identified loss of function of KIDLIA (also referred to as KIAA2022 or Xpn) at chromosome Xq13.2 as the gene responsible for severe ID and autistic behavior in several families (Cantagrel et al., 2004, 2009; Ishikawa et al., 2012; Van Maldergem et al., 2013; Charzewska et al., 2014; Kuroda et al., 2015). We have previously shown that knockdown of KIDLIA in rat hippocampal neurons led to impaired neurite outgrowth (Van Maldergem et al., 2013). Patients with a loss of KIDLIA show symptoms typical of ASD including febrile seizures, repetitive behaviors, impaired language, microcephaly, and strabismus (Cantagrel et al., 2004, 2009; Van Maldergem et al., 2013), establishing the gene as a causal factor for ASD with ID.

Accumulating evidence has shown that alterations in neurite outgrowth and branching are a common phenotype in neurodevelopmental disorders including ID and ASD (DiCicco-Bloom et al., 2006; Zikopoulos and Barbas, 2010). Many autism-related mutations, such as those in phosphatase and tensin homolog on chromosome 10, tuberous sclerosis complex 1, or SHANK3, result in an excess of branching (Kwon et al., 2006; Peça et al., 2011; Weston et al., 2014). Others, such as mutations in methyl CpG binding protein 2, thousand and one kinase 2, and endosomal Na^+^/H^+^ exchanger 6, lead to diminished branching (Belichenko et al., 2009; de Anda et al., 2012; Ouyang et al., 2013). As KIDLIA is a novel gene product involved in autism with severe ID, its role in brain development including neurogenesis, neuron migration, and neuron morphogenesis has not been investigated. Our previous work has suggested an involvement of KIDLIA in neurite outgrowth (Van Maldergem et al., 2013), but the molecular mechanisms remain unknown.

N-cadherin–mediated extracellular interactions have been shown to be required for dendrite growth (Tan et al., 2010). A major molecule that interacts with the cytoplasmic C terminus of N-cadherin is δ-catenin. δ-Catenin, a member of the p120 catenin family, is neuron specific and binds to the juxtamembrane segment of N-cadherin (Lu et al., 1999). δ-Catenin is a major candidate gene in autism and part of a protein network that is strongly involved in regulating dendrite growth (Turner et al., 2015). Overexpression of δ-catenin can induce dendritic protrusions in hippocampal neurons (Martinez et al., 2003), whereas loss of δ-catenin reduces dendritic growth and branching (Elia et al., 2006; Arikkath et al., 2008).

For the first time, we report a role for KIDLIA in neuron migration and dendrite morphological development. We show that KIDLIA is expressed exclusively in the nucleus and is neuron specific. We find that loss of KIDLIA produces aberrant neuronal migration with major defects in apical dendrite growth and orientation *in vivo*. Knockdown of KIDLIA *in vitro* results in a decrease in dendritic growth and actin dynamics. A loss of KIDLIA also leads to increased N-cadherin at the plasma membrane and an elevated interaction between N-cadherin and δ-catenin. This increased association depletes the cytoplasmic pool of δ-catenin, causing activation of RhoA-GTP. Consistent with this, we find that after KIDLIA knockdown in neurons, overexpressing δ-catenin or inhibiting RhoA activity rescues the defects in dendritic growth and actin dynamics. These findings strongly support a role for the N-cadherin/δ-catenin–RhoA signaling system in the KIDLIA-dependent dysregulation of dendritic morphogenesis, providing novel insights into the mechanism of KIDLIA-dependent autism and ID.

## Materials and Methods

### Antibodies, plasmids, and drugs

Primary antibodies to the following proteins were used: rabbit anti-KIAA2022 [1:100 (brain slice), 1:500 (primary culture) for immunohistochemistry (IHC), 1:500 for Western blot (WB), Sigma-Aldrich, St. Louis, MO; cat. # HPA000404, RRID: AB_1079208], mouse anti-NeuN (1:100 for IHC; Millipore, Billerica, MA), rabbit anti-GFAP (1:100 for IHC; Millipore; cat. # MAB377, RRID: AB_2298772), mouse anti-Tau1 (1:800 for IHC; EMD Millipore; cat. # MAB3420, RRID: AB_94855), rabbit anti-MAP2 [1:1000 for immunocytochemistry (ICC); Abcam, Cambridge, UK; cat. # ab70218, RRID: AB_1269354] mouse anti–N-cadherin [1:1000 for WB and 5 µg for immunoprecipitation (IP); BD Biosciences, Franklin Lakes, NJ; cat. # 610920, RRID: AB_2077527], mouse anti–δ-catenin (1:1000 for WB, 5 µg for IP; BD Biosciences; cat. # 611536, RRID: AB_398994), mouse anti-RhoA (1:500 for WB; Cytoskeleton, Denver, CO; cat. # ARH03-A, RRID: AB_10708069), mouse HDAC1 (1:1000 for WB; Cell Signaling Technology, Danvers, MA; cat. # 5356P, RRID: AB_10858225), mouse anti–α-tubulin (1:5000 for WB; Sigma-Aldrich; cat. # 00020911 RRID: AB_10013740), and mouse anti-GAPDH (1:3000 for WB; Abcam; cat. # ab8245 RRID: AB_2107448). The following secondary antibodies were used: immunoglobulin G/horseradish peroxidase for WB [1:5000; BioRad, Hercules, CA; mouse (cat. # 170-6516 RRID: AB_11125547) and rabbit (cat. # 170-6515 RRID: AB_11125142)], Alexa Fluor 488 (1:700, Invitrogen, San Diego, CA; mouse: cat. # A21121 RRID: AB_141514; rabbit: cat. # A11094, RRID: AB_221544), and Alexa Fluor 555 (1:700, Molecular Probes, mouse: cat. # A21127 RRID: AB_141596; rabbit: cat. # A21428 RRID: AB_141784) for ICC.

GFP–δ-catenin was a gift from S. Bamji (University of British Colombia, Vancouver), pEGFP-N1 was obtained from Addgene, Cambridge, MA (cat. # 2491, RRID: SCR_005907). For lentiviral shRNA, two KIDLIA shRNA sequences and a scrambled shRNA sequence were designed using the small interfering RNA (siRNA) Wizard v3.1 (Invivogen, San Diego, CA) and cloned into the pLKO.1-TRC cloning vector (Addgene; cat. # 10878, RRID: SCR_005907) using AgeI and EcoRI sites. For *in utero* electroporation, the same shRNA sequences were cloned into the pCGLH GFP vector using BglII and SalI sites. KIDLIA siRNA oligomers for transfection were purchased from Qiagen, Hilden, Germany. The RhoA inhibitor, CN06, was added directly to the culture media for the times indicated (10 μm; Cytoskeleton; cat. # CN06).

### Primary neuronal culture

Cortical and hippocampal brain tissue were dissected out from E18 rat fetus brains of either sex and prepared for primary culture. Tissues were first digested with papain (0.5 mg/ml in Hanks balanced salt solution, Sigma-Aldrich; cat. # 4762) at 37°C for 15 min, then gently triturated in trituration buffer [0.1% DNase (cat. # PA5-22017 RRID: AB_11153259), 1% ovomucoid (Sigma-Aldrich; cat. # T2011)/1% bovine serum albumin (Sigma-Aldrich; cat. #05470) in Dulbecco’s modified Eagle’s medium] until neurons were fully dissociated. Dissociated cortical neurons were then counted and plated into either six-well plates or 60-mm Petri dishes (Greiner Cellstar) for WB experiments. Hippocampal neurons were plated on 18-mm circular coverslips (Carolina, Burlington, NC; cat. # 633013, No. 0) in 60-mm Petri dishes (five coverslips/dish) and six-well glass-bottom imaging dishes (In Vitro Scientific, Mountain View, CA; cat. # P06-20-1-N) for ICC and fluorescence after photobleaching (FRAP) experiments. Both dishes and coverslips were coated with poly-l-lysine (Sigma-Aldrich; cat. # P2636; 100 μg/ml in borate buffer) overnight at 37°C then washed three times with sterile deionized water and left in plating medium [minimal essential medium (500 mL) containing 10% fetal bovine serum (Atlanta Biologicals, Flowery Branch, GA; cat. # S11550), 5% horse serum (Atlanta Biologicals; cat. # S12150), 31 mg l-cysteine, 1% penicillin/streptomycin (Corning, Corning, NY; cat. # 30-002-Cl), and l-glutamine (Corning; cat. # 25-005-Cl) before cell plating. Plating medium was replaced by feeding medium (neurobasal medium supplemented with 1% horse serum, 2% B-27, and 1% penicillin/streptomycin and l-glutamine) the day after cell plating. Neurons were maintained in feeding medium with 5′-fluoro-2′-deoxyuridine (10 μm; Sigma-Aldrich; cat. # F0503) supplemented at 5 d in vitro (DIV5) to suppress glial growth until experimental use. Cultures treated with virus were given 250 μl of viral medium added directly to the plating medium at time of plating. The virus was removed 18–24 h later, when feeding medium was added.

### Neuronal transfection and viral infection

Hippocampal neurons were transfected at the time of plating in six-well glass-bottom dishes for FRAP experiments using Lipofectamine 2000 (Thermo Fisher Scientific, Waltham, MA; cat. # 11668019) and the target plasmid DNA or siRNA per the manufacturer’s suggestion. For live imaging experiments, neurons were transfected at DIV5. For one well containing 2 ml plating medium + cells, 3 µl Lipofectamine 2000 and 1 µg plasmid DNA + 2 µl siRNA (20 µm) were first separately diluted in 50 µl minimal essential medium then mixed and incubated at room temperature for 20 min to form the transfection complex. The transfection complex was added to the wells and incubated at 37°C for 4 h before the medium was removed and replaced with feeding medium. Neurons were then cultured for stated times for FRAP or live imaging experiments.

Recombinant lentiviruses were produced by transfecting HEK293T cells with plasmids for the shRNA constructs with viral packaging and envelope proteins (pRSV/REV, pMDLg/RRE, and pVSVG) using polyethylenimine reagent (Polysciences, Warrington, PA; cat. # 23966). Conditioned medium containing lentivirus was harvested after 48 h, centrifuged at 1000 × *g* for 10 min, filtered through a 0.45-μm filter, and stored at −80°C. Neurons were infected with lentivirus on the day of plating, and medium was changed 1 d later to feeding medium.

### Animals

Timed pregnant CD-1 mice were purchased from Charles River Laboratories (Cambridge, MA; strain code 022) for *in utero* electroporation experiments. All animals were maintained in accordance with guidelines of the Boston University Institutional Animal Care and Use Committee. Care was taken to minimize suffering of the animals during surgical procedures. The first neonatal day was considered to be postnatal day 0 (P0).

### *In utero* electroporation

*In utero* electroporation (IUE) was performed as described previously (Gal et al., 2006) on timed pregnant CD-1 dams at embryonic day 14.5. Briefly, dams were anesthetized via i.p. injection of a ketamine/xylazine mixture, and the uterine horns were exposed via midline laparotomy. One to two microliters of plasmid DNA mixed with 0.1% fast green dye (Sigma-Aldrich; cat. # F7258) was injected intracerebrally through the uterine wall and amniotic sac using a pulled-glass micropipette. The plasmid vectors were used at a final concentration of 2–3 μg/μl. The anode of a Tweezertrode (Harvard Apparatus, Holliston, MA) was placed over the dorsal telencephalon above the uterine muscle, and four 35-V pulses (50-ms duration separated by a 950-ms interval) were applied with a BTX ECM830 pulse generator (Harvard Apparatus). After electroporation, the uterine horns were returned to the abdomen, the cavity was filled with a warm saline solution, and the incisions were closed with silk sutures. The dams were then placed in a clean cage and monitored closely during recovery. The pups were allowed to mature with the mother until the times indicated. To collect the electroporated brains, animals were anesthetized with an i.p. injection of ketamine/xylazine and transcardially perfused with ice-cold PBS. The brains were removed and placed into 4% paraformaldehyde in PBS solution at 4°C for 6 h, followed by overnight incubation in a 30% sucrose PBS solution at 4°C. The brains were placed in trays and submerged in optimum cutting temperature embedding medium (Tissue-Tek; cat. # 25608-930) and flash frozen by placing the trays in a bath of methanol mixed with dry ice. Frozen brains were cut in 35-μm sections on a Leica CM 1850 cryostat (Leica Biosystems, Buffalo Grove, IL) at –20°C. These procedures were reviewed and approved by the Boston University Institutional Animal Care and Use Committee.

### Immunocytochemistry

Hippocampal neurons were washed twice in ice-cold artificial cerebrospinal fluid (ACSF) and fixed for 10 min in a 4% paraformaldehyde/4% sucrose solution at room temperature. Cell membranes were permeabilized for 10 min in 0.3% Triton X-100 (Sigma-Aldrich; cat. # T-9284) in PBS, rinsed three times in PBS, then subjected to a blocking procedure (1 h PBS + 5% goat serum). After blocking, cells were incubated with primary antibodies (in 5% goat serum and PBS) for 2 h at room temperature, washed, and incubated with Alexa Fluor–conjugated fluorescent secondary antibodies (1:700) for an additional hour. Cells were then mounted to microscopy glass slides with Prolong Gold anti-fade mounting reagent (Thermo Fisher Scientific; cat. # P36930) for visualization.

### Microscopy

For *in vitro* analysis of neuronal morphology, a Zeiss inverted fluorescent microscope was used to collect images with a 40× oil-immersion objective [numerical aperture (NA) 1.3] and collected with AxioVision Release 4.5 software. Neuron images were quantified in Sholl analysis using NIH ImageJ (see below).

Brain sections taken after IUE were imaged using a Zeiss LSM 700 laser scanning confocal microscope. *Z*-stack images (25 μm, 40×) were acquired using the Zen software package. GFP-positive cells were traced with the NeuronJ plugin in ImageJ for analysis of dendrite morphology. Sections from at least three embryos were counted and analyzed for each experiment, and surgeries for each combination of plasmids were repeated to confirm the results.

### Sholl analysis

MAP2-positive dendrites were traced from images of neurons stained with MAP2 and tau1 using the NeuronJ (RRID: SCR_002074) plugin in ImageJ (RRID: SCR_003070). The Snapshot tool in NeuronJ was used to save the tracings as an image file that was converted to 8-bit, and these images were analyzed with the Sholl analysis plugin in ImageJ. The range of measurement was set using the straight line tool traced from the center of the soma to the outermost neurite. Dendrite intersections were analyzed from a starting radius of 10 μm with 10-μm steps to the outer radius. The resulting numbers of intersections per cell were used to calculate the mean and SEM for each radius interval. Treatments were applied to sister cultures that originated from the same neuron culture preparation.

### Western blot

Cortical neurons cultured in either 60 mm dishes (3 × 10^6^ cells/dish) or six-well plates (10^6^ cells/well) were treated with virus at the time of plating and any other drugs as stated. Neurons were lysed in Laemmli 2× sample buffer (4% SDS, 10% 2-mercaptoethanol, 20% glycerol, 0.004% bromophenol blue, and 0.125 m Tris-HCl) and boiled for 10 min at 95°C for SDS-PAGE electrophoresis. After separation in SDS-PAGE, proteins were transferred to polyvinylidene fluoride membranes (Bio-Rad, Richmond, CA) and probed for different targets with the stated antibodies. Immunoblots were visualized using a chemiluminescence detection system (GE Healthcare, Piscataway, NJ), exposed to Fuji medical X-ray films (Thermo Fisher Scientific), scanned, and analyzed using ImageJ.

### Quantitative real-time PCR

Total RNA from rat cortical cultures was purified using TRIzol (Thermo Fisher Scientific; cat. # 12183555) and reverse-transcribed using the SuperScript III Reverse Transcription System (Thermo Fisher Scientific; cat. # 12574035). The optical density A260/A280 ratio was confirmed to be >1.9 for each sample. For N-cadherin, we used the following oligonucleotides: 5′-ATCATTCGCCAAGAGGAAGG-3′ and 5′-GGCTGAAAATAGACCCTGTGA-3′. Quantitative real-time PCR was performed with a 7300 real-time PCR system (Applied Biosystems, Foster City, CA) using Power SYBR Green Master Mix (Applied Biosystems; cat. # 4367659) with the following PCR conditions: initial hold at 95°C for 10 min, followed by 40 cycles of a 15-s denaturing step at 95°C and a 60-s annealing and extension step at 60°C. Transcript levels were normalized to the housekeeping gene GAPDH using the following oligonucleotides: 5′-CCATCAACGACCCCTTCATT-3′ and 5′-CTGAGAATGGGAAGCTGGTC-3′.

### Cell-surface protein biotinylation assay

Cortical neurons were rinsed with ACSF once and incubated with EZ Link-Sulfo-NHS-LC-Biotin (Thermo Fisher Scientific; cat. # 21327) dissolved in 1 ACSF (1 mg/ml) for 10 min at room temperature and another 20 min at 4°C. Excess biotin reagent was quenched with two washes of ACSF with 20 mm glycine followed by another two washes with ACSF. Neurons were then lysed in lysis buffer 1 (1× PBS with 0.5% SDS, 0.5% sodium deoxycholate, and 1% Triton X-100) with the addition of a protease inhibitor cocktail (Roche, Basel, Switzerland; cat. # 04693116001), sonicated, then head-to-toe rotated at 4°C for 30 min to achieve thorough cell lysis. After spinning down at 13,000 rpm for 15 min, one tenth of the supernatant volume was mixed with an equal volume of Laemmli 2× sample buffer, boiled for 10 min at 95°C, and saved as a total lysate control. The rest of the supernatant was removed to a new Eppendorf 1.5-ml tube containing 40 μl of pre-equilibrated NeutrAvidin beads (Thermo Fisher Scientific; cat. # 29200), and the pellet was discarded. NeutrAvidin beads, along with the supernatant, were head-to-toe rotated at 4°C for at least 2 h. The beads were rinsed three times in PBS (with 0.5% Triton X-100), before being mixed with an equal volume of Laemmli 2× sample buffer and boiled for 10 min at 95°C in preparation for WB analysis.

### Fluorescence after photobleaching

Images were taken with a Zeiss LSM 700 laser scanning confocal microscope. The inverted microscope was equipped with an incubation system featuring temperature and CO_2_ control. All experiments were performed at 37°C and 5% CO_2_. Live images were acquired using a 63× oil-immersion objective lens (NA 1.32).

FRAP experiments were performed using the following protocol: three single prebleach scans were acquired at 3-s intervals, followed by up to five bleach scans at full laser power until the fluorescence reached 25% of the original prebleach levels, over a circular area of 4 μm in diameter. During the postbleach period, scans were acquired at 3-s intervals.

Fluorescence was quantified using the FRAP plugin in ImageJ. Background fluorescence was measured in a random field outside of the region of interest (ROI) and subtracted from all the measurements. Growth cone fluorescence was determined for each image and normalized to the initial prebleach fluorescence to determine the rate of fluorescence recovery.

The net fluorescence recovery (mobile fraction, *M_f_*) measured in the region of interest was determined as *M_f_* = (*F_pre_* – *F_post_*) – (*F_pre_* – *F_end_*), and the immobile fraction (*IM_f_*) was calculated as *IM_f_* = (*F_pre_* – *F_post_*) – (*F_end_* – *F_post_*), where *F_end_* is the ROI mean intensity at steady state, *F_post_* is ROI intensity postbleach, and *F_pre_* is mean ROI intensity prebleach.

### RhoA activation assay

RhoA activity was assessed using a RhoA Activation Assay Biochem Kit according to the manufacturer’s instructions (Cytoskeleton; cat. # BK036). Briefly, GTP-RhoA was immunoprecipitated from whole-cell lysates with glutathione S-transferase–tagged Rhotekin bound to glutathione agarose beads. The beads were washed, and immunoprecipitates were analyzed by WB using a RhoA-specific monoclonal antibody. The lysate was also probed for total RhoA, and GTP-RhoA was normalized to total RhoA levels.

### Statistical analyses

An unpaired Student’s *t*-test or one-way ANOVA with post hoc Tukey’s test was used as appropriate.

## Results

### KIDLIA shows nuclear localization and neuron-specific expression *in vivo*

**Figure 1.**
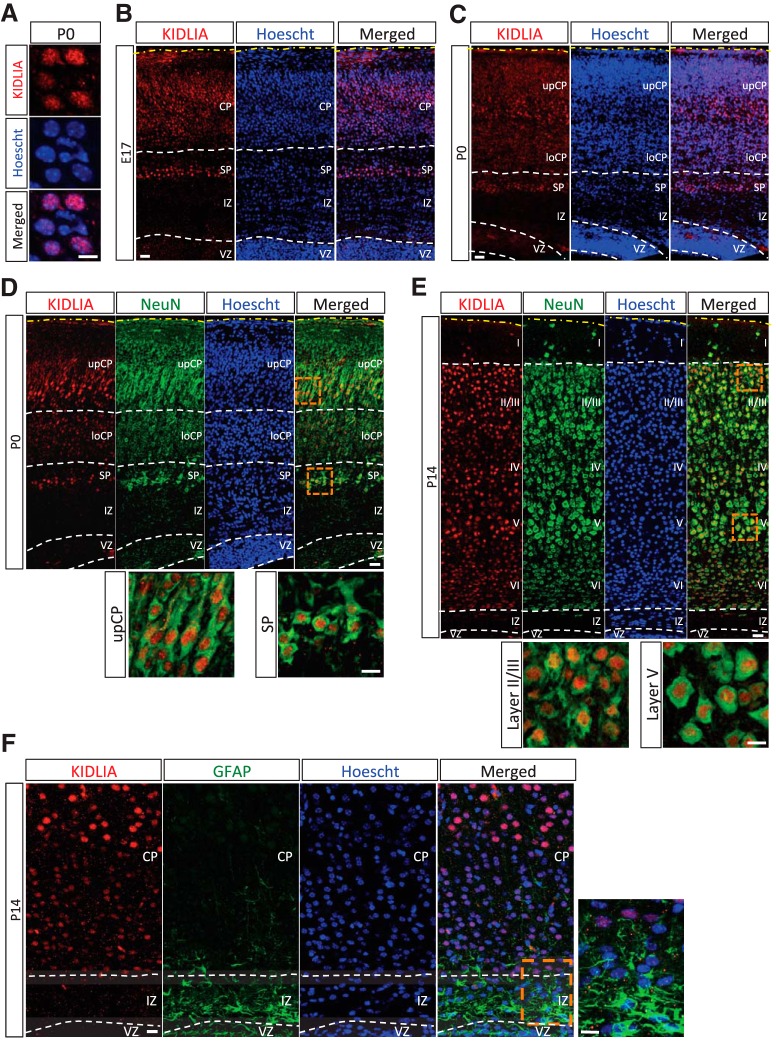
KIDLIA is expressed in neurons and is localized in the nucleus. ***A***, Immunohistochemistry in brain slices of P0 mouse cortex. KIDLIA was colocalized with the nuclear marker Hoechst. Scale bar = 10 μm. ***B***, ***C***, Immunohistochemistry of KIDLIA at E17. KIDLIA expression began in the SP region of the upper IZ and throughout the cortical plate and was restricted to the cortical plate at P0. Scale bars = 20 μm. ***D***, ***E***, KIDLIA was expressed only in cells positive for the neuronal marker NeuN at P0 (left) and P14 (right). ***F***, KIDLIA expression was not observed in cells expressing the glia marker GFAP at P14. Scale bars = 20 μm (full picture); 10 μm (enlarged area). UpCP, upper cortical plate; LoCP, lower cortical plate.

*In situ* hybridization studies have shown a lack of KIDLIA mRNA in proliferating, bromodeoxyuridine-positive cells, indicating that it may be expressed in postmitotic neurons only (Cantagrel et al., 2009); however, KIDLIA protein expression has not been examined in neurons. By immunostaining KIDLIA in brain slices from P0 (day of birth) mouse cortex, we observed an exclusive nuclear localization of KIDLIA that colocalized with the nuclear marker Hoechst (Fig. 1*A*). To further investigate the KIDLIA expression pattern in cortical layers *in vivo*, we compared the KIDLIA distribution in embryonic day 17 (E17) and P0 mouse brains. In E17 brain slices, KIDLIA was not expressed in the ventricular zone (VZ) or intermediate zone (IZ). Up from the VZ, only minimal background staining of KIDLIA protein was detected until the subplate (SP) region, where KIDLIA expression levels were sharply increased and maintained throughout the entire cortical plate (CP) (Fig. 1*B*). Similarly, immunostaining of P0 brains showed that strong KIDLIA expression was restricted to the CP in contrast to the VZ/IZ area (Fig. 1*C*). To determine whether KIDLIA was preferentially expressed between neurons and glia, we coimmunostained KIDLIA with the neuron-specific marker NeuN or the glial marker GFAP. We found that immunofluorescent signals of KIDLIA completely colocalized with NeuN-positive neurons at P0 and P14 (Fig. 1*D*, *E*), but not with GFAP-positive glia (P14; Fig. 1*F*), indicating that KIDLIA expression was neuron specific.

### *In utero* knockdown of KIDLIA regulates neuronal migration without affecting the multipolar-to-bipolar transition

**Figure 2.**
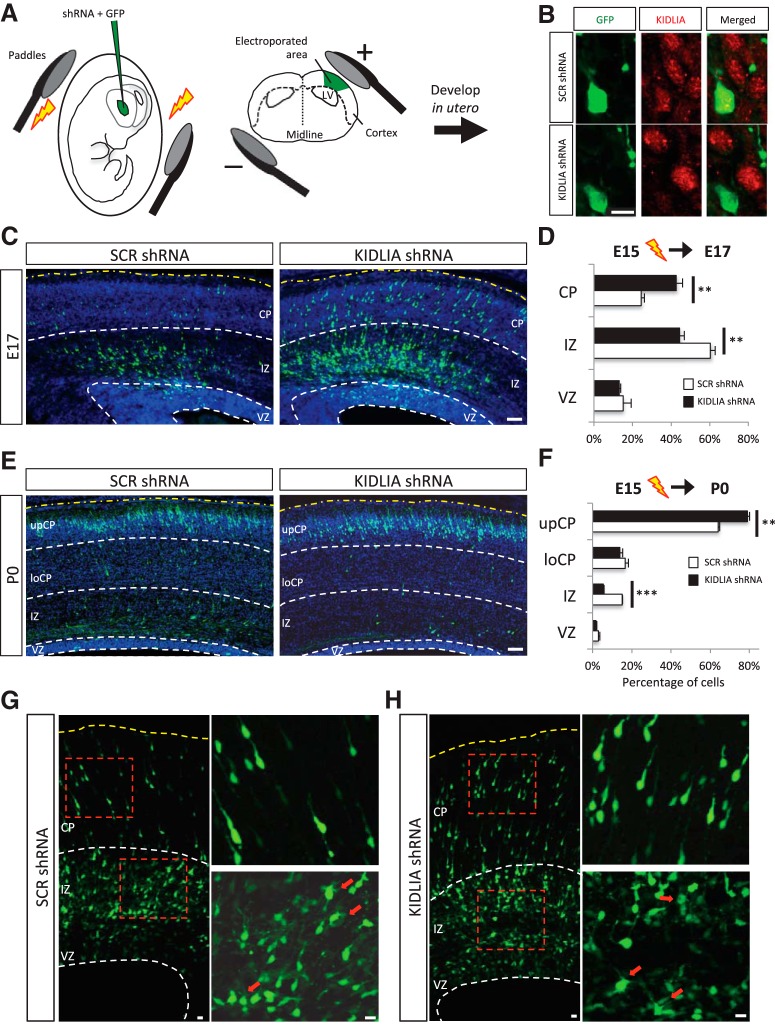
*In utero* electroporation of KIDLIA shRNA disrupts neuronal migration but does not affect the multipolar-to-bipolar transition. ***A***, Schematic of the IUE procedure. Pups were injected with shRNA-GFP DNA into the lateral ventricle (LV) at E15, and the anode of the Tweezertrode was placed above the dorsal telencephalon. Pups were the returned to the mother to mature until the times indicated. ***B***, Representative images of scrambled and KIDLIA shRNA electroporated neurons at E17. Immunostaining of KIDLIA shows a clear reduction of KIDLIA expression compared with scrambled controls and nearby nonelectroporated neurons. Scale bar = 10 μm. ***C***, Brain slices taken at E17 after IUE of KIDLIA shRNA-GFP or scrambled shRNA-GFP at E15. Scale bar = 50 μm. ***D***, KIDLIA knockdown caused a greater percentage of neurons in the upCP and a smaller fraction in the IZ. More than 1500 GFP^+^ neurons from five brains were analyzed in each group. ***E***, Brain slices taken at P0 after IUE of KIDLIA shRNA-GFP or scrambled shRNA-GFP at E15. Scale bar = 50 μm. ***F***, Analysis of neuronal migration at P0 showed that more neurons were in the upCP and less in the IZ compared with scrambled controls. More than 1200 GFP^+^ neurons from four brains were analyzed in each group. ***G***, ***H***, Analysis of the multipolar-to-bipolar transition. In E17 brain, multipolar neurons were observed in the upper IZ (magnified lower right) before CP entry, and neurons showed a normal transition back to a bipolar morphology after CP entry (magnified upper right) in both KIDLIA and scrambled shRNA electroporated neurons. Scale bars = 10 μm. **p < 0.01, ***p < 0.001. Error bars = SEM. Yellow dashed line indicates the pia. UpCP, upper cortical plate; LoCP, lower cortical plate.

To investigate KIDLIA’s role in neuronal migration, we used IUE to knock down KIDLIA expression during early development. KIDLIA shRNA-GFP or a scrambled control was electroporated into mouse embryonic brains at E15 and returned to the pregnant dam to develop (Fig. 2*A*). Brains were first collected at E17 and immunostained for KIDLIA. In GFP-positive shRNA electroporated neurons, KIDLIA expression was significantly reduced compared with scrambled controls and nearby nonelectroporated neurons (Fig. 2*B*).

Callosal projecting pyramidal neurons are born around E15 in the VZ and migrate to their final location in layers II/III in the CP. At E17, 60% of the neurons electroporated with scrambled shRNA were found in the IZ, and only 13% had entered the CP. Strikingly, after electroporation of KIDLIA shRNA, 44% of the neurons were found in the IZ and 28% of the neurons had already migrated into the CP (Fig. 2*C*, *D*). These results strongly indicate that loss of KIDLIA expression alters the migration process in the developing brain.

To investigate the effect of KIDLIA on neuron migration at a later stage, we next collected brains at P0 after IUE at E15. In agreement with our findings at E17, we found that 79% of KIDLIA-shRNA neurons had already reached the upper CP at P0, compared with only 64% in scrambled controls. Additionally, in shRNA electroporated brains, only 5% of neurons were found in the IZ compared with 15% in scrambled control brains (Fig. 2*E*, *F*). Interestingly, although KIDLIA knockdown resulted in changes in the relative layer distribution of the migrating neurons, it did not affect their final laminar destination, with the majority of the electroporated neurons residing in layer II/III (Fig. 2*E*).

After birth in the VZ, multipolar pyramidal neurons move within the IZ/SP, where they must adopt a bipolar morphology to enter the CP. This multipolar-to-bipolar transition is key to proper migration and integration of neurons into the cortical plate. KIDLIA proteins begin to be expressed in the SP region below the cortical plate at E17 and may be involved in neuronal migration. We therefore wanted to know whether loss of KIDLIA expression resulted in neurons bypassing the multipolar stage, allowing them to move into the CP directly. In mice electroporated with KIDLIA shRNA at E15, we found that in E17 brains both KIDLIA shRNA and scrambled control neurons showed a typical multipolar morphology in the IZ/SP region. Similar to controls, KIDLIA knockdown neurons showed a normal bipolar morphology after entering the CP (Fig. 2*G*, *H*). These findings suggest that KIDLIA does not affect multipolar-to-bipolar transition during neuronal migration.

### Knockdown of KIDLIA affects apical dendrite orientation *in vivo*

**Figure 3.**
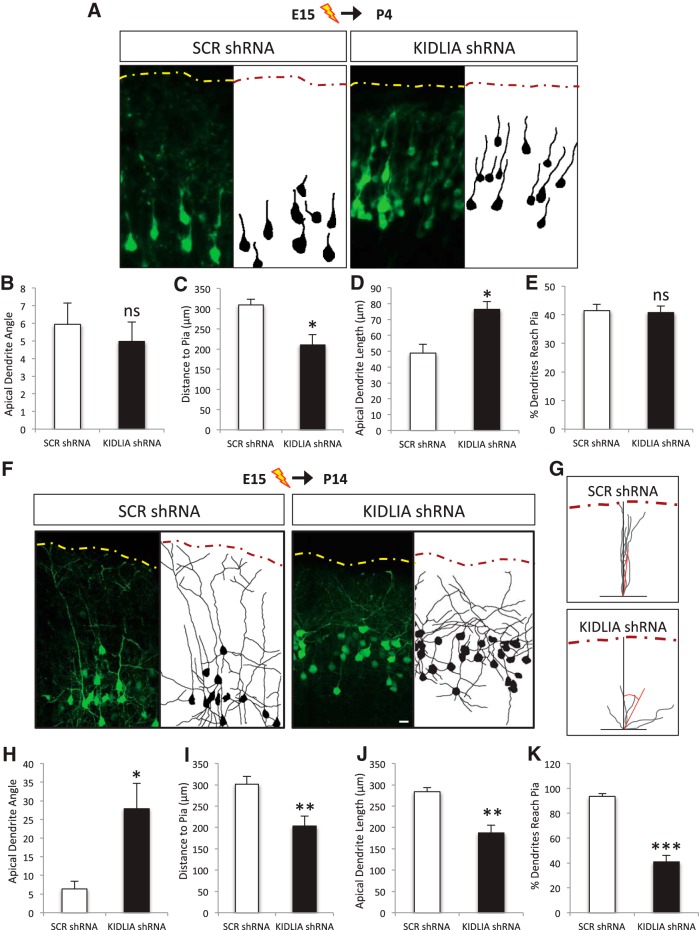
Knockdown of KIDLIA affects apical dendrite growth and orientation *in vivo.* ***A***, Images (left) and tracings (right) of P4 layer II/III cortical neurons after electroporation of GFP-labeled shRNA constructs at E15. Yellow dashed lines indicate the pia; scale bar = 10 μm. ***B***, No significant change was observed in the apical dendrite angle to the pia after knockdown of KIDLIA. ***C***, An increase was observed in the length of the major apical dendrite (*n* = 6). ***D***, Decrease in the distance of the soma to the pia (*n* = 6). ***E***, No significant difference was observed in the number of dendrite tips reaching the pia. ***F***, Images (left) and tracings (right) of P14 layer II/III cortical neurons after electroporation of GFP-labeled shRNA constructs at E15.5. Dashed lines indicate the pia; scale bar = 10 μm. ***G***, Merged tracings of the major apical dendrites of P14 neurons after IUE of scrambled and KIDLIA shRNA showed that knockdown of KIDLIA disrupted the orientation of the apical dendrites toward the pia. Red bars show the average angle of the dendrites in relation to the pial surface (dashed line). ***H***, Quantification of the angle of the major apical dendrite toward the pia showed a significant increase after loss of KIDLIA expression (*n* = 5). An angle of 0° indicates that a dendrite is growing directly toward the pia. ***I***, A significant decrease in the length of the major apical dendrite. ***J***, A decrease in the distance of the soma to pia. ***K***, A significant decrease in the percentage of dendrite tips that reached the pia was observed after knockdown of KIDLIA *in vivo*. *p < 0.05, **p < 0.01, ***p < 0.001. Error bars = SEM.

To examine the role of KIDLIA in neuronal development, we analyzed dendritic growth and soma location of neurons after IUE of KIDLIA shRNA at E15. At P4, neurons have reached their proper laminar location at layer II/III, but the somas of neurons electroporated with KIDLIA-shRNA were located closer to the pial surface (Fig. 3*A*, *C*), possibly as a result of a facilitated migration rate or a disruption in the termination of migration. Similar to scrambled controls, neurons with KIDLIA shRNA showed a clear apical dendrite growing as single straight process directed orthogonally to the pial surface (Fig. 3A, *B*). However, neurons expressing KIDLIA shRNA had a longer apical neurites (Fig. 3*A*, *D*). The percentage of cells whose apical neurites reached the pia was not significantly different from controls at P4 (Fig. 3*E*).

Strikingly, brains analyzed at P14 showed that electroporation of KIDLIA shRNA significantly disrupted the orientation of the apical dendrite. The apical dendrites of neurons after KIDLIA knockdown were largely disorganized and not directed toward the pia, compared with age-matched controls (Fig. 3*F*, *G*). A significant increase and variation in the angle of the apical dendrite to the pia was observed in KIDLIA shRNA electroporated brains compared with scrambled controls (Fig. 3*H*). KIDLIA knockdown also produced a 35% decrease in the length of the major apical dendrite and a significant decrease in the distance of their somas to the pia (Fig. 3*I*, *J*). Additionally, after KIDLIA knockdown, the number of dendrite tips that reached the pia was significantly decreased compared with controls (Fig. 3*K*), presumably owing to a disorganization of the apical dendrite orientation and decreased dendritic growth. These *in vivo* results showed that after knockdown of KIDLIA, neuronal somas were positioned at sites closer to the pial surface at both P4 and P14. A loss of KIDLIA caused aberrant dendrite orientation and decreased apical dendrite growth, indicating that the directed outgrowth and branching of the dendritic tree was significantly impaired, possibly as a consequence of the neurons’ early arrival into the CP or a subtle shift in laminar localization.

### Knockdown of KIDLIA decreases dendritic outgrowth in primary cultured neurons

**Figure 4.**
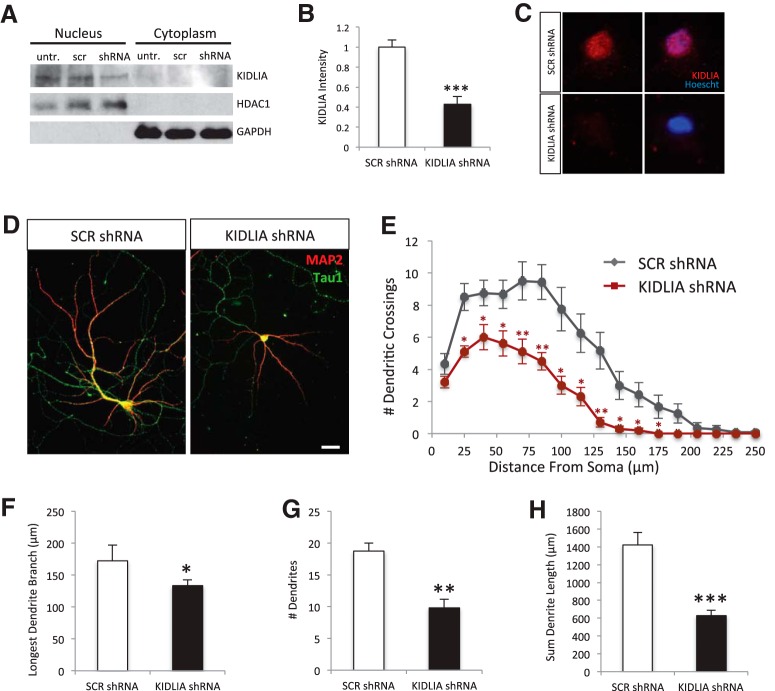
Knockdown of KIDLIA decreases dendritic growth and branching *in vitro.* ***A***, Western blot after nuclear/cytoplasmic fractionation of primary rat hippocampal neurons shows clear nuclear expression of KIDLIA with no expression in cytoplasm. Neurons were treated with lentiviral KIDLIA shRNA or scrambled shRNA at DIV0 and collected at DIV6. ***B***, Analysis of the Western blot data showed that the shRNA significantly reduced KIDLIA expression (*n* = 5). Nuclear loading control, HDAC1; cytoplasmic loading control, GAPDH. ***C***, Immunostaining of KIDLIA in primary rat hippocampal neurons shows colocalization with the nuclear marker Hoechst. ***D***, Immunostaining of dendrites (MAP2) and axons (tau1) at DIV12; scale bar = 10 μm. ***E***, Sholl analysis of dendrite growth at DIV12 showed a significant change in branching (*n* = 14). ***F***, Analysis of MAP2-positive dendrites showed a decrease in the longest dendrite segment (*n* = 14). ***G***, Decrease in the number of dendrite branches (*n* = 14). **(H)** Significant decrease in the sum dendritic length (*n* = 14). *p < 0.05, **p < 0.01, ***p < 0.001. Error bars = SEM.

To further investigate the molecular mechanisms underlying the effect of KIDLIA on dendritic growth and arborization, we suppressed KIDLIA expression in primary cultured neurons. We infected cortical neurons at the time of plating (DIV0) with lentiviral shRNA containing the same targeting sequences as those used for IUE *in vivo* and found a ∼60% reduction in KIDLIA protein expression 6 d after infection (Fig. 4*A*, *B*). Consistent with the *in vivo* observations, we confirmed a similar nuclear localization of KIDLIA in cultured neurons (Fig. 4*A*, *C*).

To investigate the effect of KIDLIA knockdown on dendritic outgrowth, we infected neurons with KIDLIA shRNA virus at DIV0 and immunostained for MAP2 and tau1 at DIV12 to label the dendrite and axon, respectively (Fig. 4*D*). Strikingly, Sholl analysis revealed a large decrease in dendritic branching after KIDLIA knockdown (Fig. 4*E*). Compared with scrambled controls, we observed a ∼25% reduction in the length of the longest dendrite, a nearly 50% reduction in the number of dendritic branches, and a 55% reduction in total dendritic length (Fig. 4*F–H*). These results show that loss of KIDLIA expression severely stunted neuronal development *in vitro*.

### Knockdown of KIDLIA leads to an increase in surface N-cadherin/δ-catenin association

N-cadherin, the neural member of the cadherin superfamily, has been shown to be involved in neurite outgrowth (Bard et al., 2008). N-cadherin is structurally composed of five extracellular cadherin domains, a single-pass transmembrane domain, and an intracellular domain that interacts with catenins that regulate downstream signaling cascades including the Rho GTPases.

**Figure 5.**
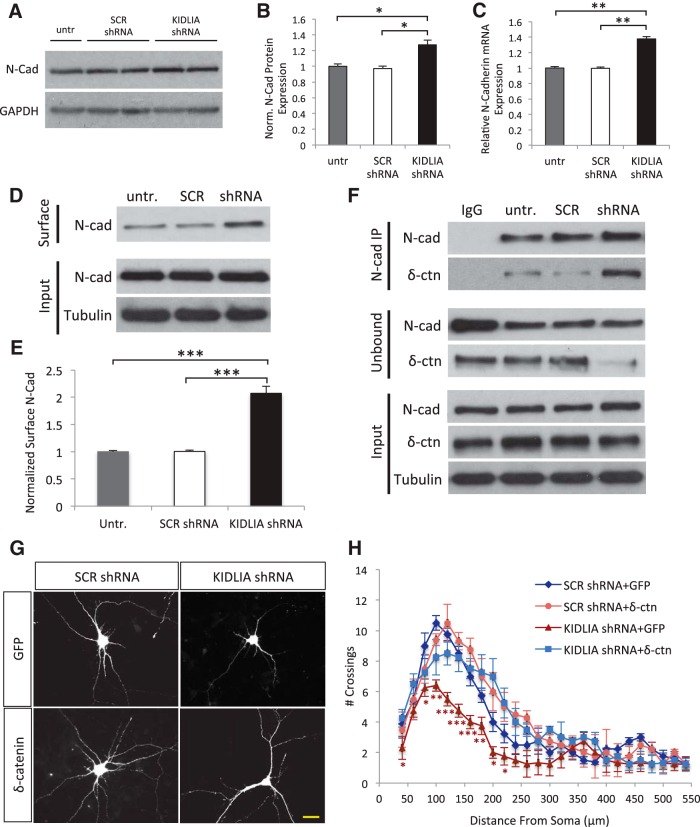
Knockdown of KIDLIA leads to an increase in surface N-cadherin and the N-cadherin/δ-catenin association. ***A***, Western blot from DIV6 neuronal lysates after treatment with KIDLIA shRNA virus (DIV0) showing an increase in total N-cadherin levels. Loading control, GAPDH. ***B***, Quantification of the Western blot data represented in ***A*** (*n* = 3, each sample performed in duplicate and averaged). ***C***, Knockdown of KIDLIA expression by shRNA lentivirus caused an increase in N-cadherin mRNA expression. Gene expression was normalized to the housekeeping gene, GAPDH (*n* = 3, each sample performed in triplicate and averaged). ***D***, Surface biotinylation of neuronal lysates after treatment with scrambled or KIDLIA shRNA virus showed an increase in surface N-cadherin levels. ***E***, Quantification of the Western blot data shown in ***C*** (*n* = 4). ***F***, N-cadherin was co-immunoprecipitated with a dramatically larger fraction of δ-catenin after lentiviral shRNA knockdown of KIDLIA in neuronal lysates. The increased binding of δ-catenin to N-cadherin subsequently depleted the unbound, cytosolic fraction of δ-catenin. ***G***, Images of neurons transfected with scrambled or KIDLIA shRNA with either GFP or δ-catenin–GFP; scale bar = 10 μm. ***H***, Sholl analysis of the transfected neurons from ***E*** showed that δ-catenin overexpression could rescue the decreased dendrite growth and branching observed after knockdown of KIDLIA (*n* = 10). *p < 0.05, **p < 0.01, ***p < 0.001 Error bars, SEM.

A previous study indicated that in PC12 cells, knockdown of KIDLIA upregulates the expression of N-cadherin (Magome et al., 2013). We therefore sought to investigate whether knockdown of KIDLIA affected N-cadherin expression and localization in neurons. In DIV6 neurons that were infected with KIDLIA shRNA at DIV0, we found that KIDLIA knockdown led to a ∼25% increase in N-cadherin protein levels compared with scrambled controls (Fig. 5*A*, *B*). Because KIDLIA is localized in the nucleus, we next examined whether KIDLIA regulates N-cadherin gene transcription. We infected DIV0 cultured cortical neurons with KIDLIA shRNAs for 7 d and measured N-cadherin mRNA by RT-PCR using the cell lysates. After knockdown of KIDLIA, we found a 35% increase in N-cadherin mRNA compared with scrambled shRNA and untreated controls (Fig. 5*C*). These data suggest that the KIDLIA-induced increase in N-cadherin protein amount may result from an upregulation in its gene transcription and translation.

To assess the membrane localization of N-cadherin after KIDLIA knockdown, we isolated cell-surface proteins through biotinylation and immunoprecipitation with sulfo-NHS-SS-biotin. Surprisingly, we found a greater than twofold increase in surface N-cadherin levels after shRNA-mediated knockdown of KIDLIA compared with scrambled controls (Fig. 5*D*, *E*), indicating that KIDLIA knockdown not only increased overall N-cadherin expression, but also caused a translocation from the cytosolic compartment to the cell surface.

δ-Catenin is a critical functional mediator for N-cadherin via a direct protein association. δ-Catenin expression can induce a dendritic-like morphology in fibroblasts (Yu and Malenka, 2003) and stimulates neurite outgrowth in hippocampal neurons (Martinez et al., 2003). Importantly, an increase in membrane-localized N-cadherin suppresses the growth-promoting effects of δ-catenin, suggesting that surface N-cadherin acts as a buffer regulating cytosolic δ-catenin availability (Kim et al., 2008). Given that δ-catenin is brain specific (Abu-Elneel et al., 2008), and that its localization and ability to induce neurite outgrowth are tightly linked with N-cadherin, we hypothesized that the increased membrane expression of N-cadherin sequesters δ-catenin at the surface and thus depletes its cytosolic pool.

To investigate this hypothesis, we performed coimmunoprecipitation studies using lysates of DIV6 cultured cortical neurons infected with viral KIDLIA shRNAs. We found that knockdown of KIDLIA significantly enhanced the association of N-cadherin with δ-catenin. Meanwhile, we observed a major reduction in the amount of free unbound δ-catenin in the supernatant after immunoprecipitation (Fig. 5*F*). These results indicate that loss of KIDLIA results in an increase in the association of N-cadherin with δ-catenin at the plasma membrane, resulting in a reduction in free cytosolic δ-catenin.

### δ-Catenin is involved in KIDLIA-dependent changes in neurite growth and arborization

Because free cytosolic δ-catenin is required for its downstream signaling, we next examined whether overexpression of δ-catenin after loss of KIDLIA could rescue the deficits in neurite outgrowth and branching. Neurons were treated with scrambled or KIDLIA lentiviral shRNA at DIV0 and transfected with δ-catenin–GFP or GFP control 1 d later (Fig. 5*G*). At DIV6, Sholl analysis revealed a large decrease in dendritic branching in shRNA + GFP cells compared with scrambled + GFP. However, in neurons overexpressing δ-catenin–GFP + shRNA, neurite branching was restored to levels similar to scrambled controls (Fig. 5*H*). These results strongly indicate that the decrease in neurite outgrowth and branching after knockdown of KIDLIA was caused by a reduction in free δ-catenin availability.

### Loss of KIDLIA disrupts actin dynamics at the neurite growth cone

**Figure 6.**
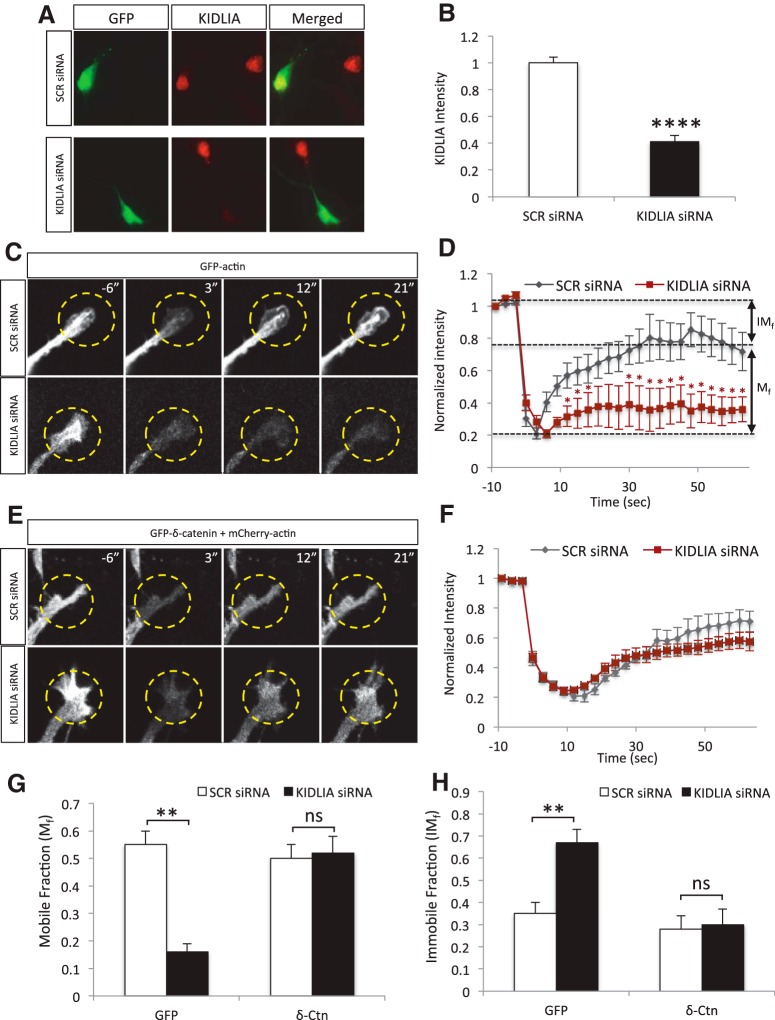
Loss of KIDLIA disrupts actin dynamics at the neurite growth cone via δ-catenin. ***A***, Immunocytochemistry of primary rat hippocampal neurons after transfection of scrambled or KIDLIA siRNA with GFP. Neurons were transfected at DIV0 and immunostained for KIDLIA (red) at DIV4. ***B***, Analysis of the immunocytochemistry images showed that the siRNA significantly reduced KIDLIA expression (*n* = 10 for both groups). ***C***, FRAP experiments after cotransfection of KIDLIA siRNA or scrambled siRNA with actin-GFP. Regions at the growing neurite tip were selected for photobleaching at 488 nm and imaged every 3 s. ***D***, Analysis of the FRAP data showed that knockdown of KIDLIA produced a dramatic decrease in actin dynamics after photobleaching (*n* = 9). ***E***, Neurons were cotransfected with KIDLIA siRNA or scrambled siRNA with actin-mCherry and δ-catenin–GFP or GFP alone, photobleached at 555 nm, and imaged every 3 s. ***F***, Analysis of the FRAP data. Overexpression of δ-catenin rescued the actin dynamics after knockdown of KIDLIA (*n* = 7). ***G***, The mobile fraction, calculated as the difference between the average level of bleaching and the level of recovery, was significantly decreased after knockdown of KIDLIA (*n* = 7). ***H***, The immobile fraction of actin, calculated as the difference between the initial fluorescence and the level of recovery, was significantly decreased (*n* = 7). **p < 0.01, ****p < 0.0001. Error bars = SEM.

Actin is dynamically regulated at the neurite tips, which plays a crucial role in neurite growth (Meberg and Bamburg, 2000; Nicholson-Dykstra et al., 2005). To examine whether KIDLIA is implicated in cytoskeletal dynamics, we performed FRAP experiments with fluorescently labeled actin after knockdown of KIDLIA with siRNA. To test the efficacy of the KIDLIA knockdown, scrambled or KIDLIA siRNA was cotransfected with GFP at DIV0, and immunostainings of KIDLIA at DIV4 showed a ∼60% reduction in protein expression (Fig. 6*A*, *B*). Next, GFP-actin was transfected at DIV0 into hippocampal neurons with scrambled or KIDLIA siRNA, and FRAP was performed on the growth cones at DIV4 (Fig. 6*C*). In neurites from KIDLIA siRNA–transfected neurons, the recovery rate of the actin signal was drastically reduced (Fig. 6*D*). Compared with scrambled siRNA controls, KIDLIA knockdown showed a 70% decrease in the mobile fraction (*M_f_*) of actin (Fig. 6*G*) and a subsequent increase in the immobile fraction (*IM_f_*) of actin (Fig. 6H).

Because of the large reduction of free δ-catenin in the cytosol after KIDLIA knockdown, which is known to be involved in actin regulation, we next sought to examine whether overexpression of δ-catenin could rescue actin dynamics with the transfection of δ-catenin–GFP after siRNA-mediated knockdown of KIDLIA (Fig. 6*E*). Indeed, FRAP revealed that in δ-catenin–transfected neurons, actin dynamics were restored (Fig. 6*F*) and the mobile and immobile fractions of actin returned to levels similar to those of controls (Fig. 6*G*, *H*), indicating that the impaired actin dynamics likely resulted from a reduction of free cytoplasmic δ-catenin.

### Role for RhoA in the KIDLIA-dependent impairment of actin dynamics

**Figure 7.**
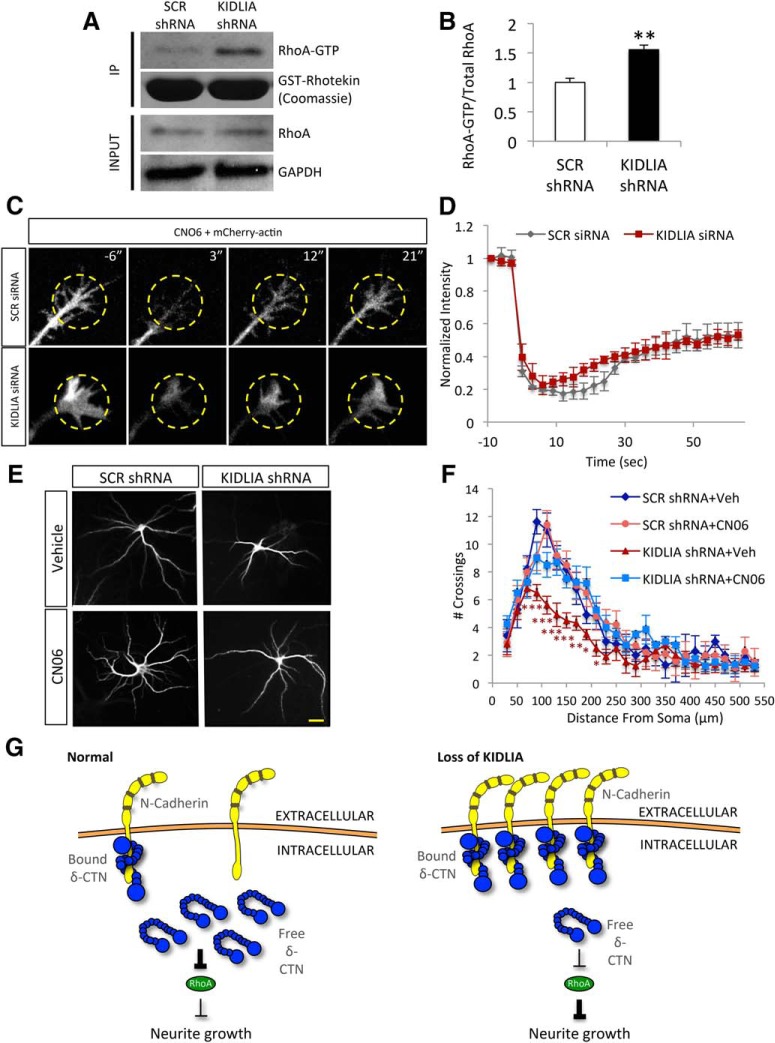
Increased RhoA activation leads to impaired actin dynamics. ***A***, Western blot at DIV6 after treatment with KIDLIA shRNA or scrambled shRNA virus at DIV0. RhoA stimulation was performed with treatment with nocodazole (10 μm) for 30 min before collection of neuronal lysates. Activated RhoA-GTP was selectively immunoprecipitated using beads conjugated to the Rho binding domain of the Rho effector protein, rhotekin, and whole-cell lysates were probed for total RhoA levels. Loading control, GAPDH. ***B***, Quantification of the RhoA assay showed a significant increase in the levels of activated RhoA-GTP after KIDLIA knockdown (*n* = 4). ***C***, Images of neurons transfected with KIDLIA siRNA or scrambled siRNA with actin-mCherry and treated with CN06 or vehicle control 1 h before FRAP experiments. ***D***, Analysis of the FRAP data showed that inhibition of the RhoA pathway could rescue the actin dynamics after knockdown of KIDLIA (*n* = 6). ***E***, Images of neurons treated from DIV4 to DIV7 with CN06 or vehicle control after treatment with KIDLIA shRNA or scrambled control virus at DIV0; scale bar = 10 μm. ***F***, Sholl analysis of the images in ***C*** showed that chronic inhibition of the RhoA pathway was sufficient to rescue dendrite outgrowth and branching (*n* = 11). ***G***, Diagram depicting changes in the N-cadherin/δ-catenin signaling cascade after loss of KIDLIA expression. Increased surface N-cadherin sequesters δ-catenin after KIDLIA knockdown, thereby depleting the cytosolic free fraction of δ-catenin. Less inhibition on RhoA increases its activation and subsequent inhibition on neurite outgrowth via changes in actin dynamics. *p < 0.05, **p < 0.01, ***p < 0.001. Error bars = SEM.

A major downstream effector of δ-catenin is the Rho-GTPase pathway, which links the cadherin/catenin complex to actin dynamics. RhoA GTPase activation leads to an inhibition in neurite growth (Kozma et al., 1997; Leeuwen et al., 1997). Studies have shown that free cytosolic δ-catenin, but not the membrane-associated fractions, inhibits RhoA activity (Kim et al., 2008). Because KIDLIA regulates the association of δ-catenin to surface N-cadherin and thus reduces the availability of cytosolic δ-catenin, we wanted to know whether KIDLIA knockdown affected RhoA activity. Rho activity status alternates between an active, GTP-bound state and an inactive, GDP-bound state. To perform RhoA activity assays, we infected neurons with lentivirus containing scrambled or KIDLIA shRNA. Neuron lysates were incubated with a GST fusion protein containing the binding domain of the Rho effector protein rhotekin, so that the active form of RhoA was isolated by immunoprecipitation. We then probed for total RhoA in lysates and the immunoprecipitated active RhoA via WB (Fig. 7*A*). Interestingly, we found that KIDLIA knockdown led to a 60% increase in the amount of active RhoA compared with scrambled controls (Fig. 7*B*). Because RhoA activation prevents neurite initiation and induces neurite retraction, our findings suggest that the inhibitory effect of KIDLIA knockdown on dendrite growth may be mediated by RhoA.

To further determine the role of RhoA activity in the KIDLIA-dependent effects on neuron development, we first performed FRAP experiments to examine the actin dynamics with the application of CN06, which directly inhibits the primary RhoA effector ROCK (Ishizaki et al., 2000). In KIDLIA knockdown neurons, application of CN06 1 h before FRAP was sufficient to rescue the actin dynamics to levels similar to those of scrambled controls (Fig. 7*C*, *D*). Furthermore, we wanted to determine whether the RhoA signaling pathway was responsible for the KIDLIA-dependent impairment in dendritic arborization. We therefore infected neurons with KIDLIA shRNA virus at DIV0 followed by CN06 treatment from DIV4 to DIV7. Dendritic structure indicated by MAP2 staining was examined via Sholl analysis. Indeed, after KIDLIA knockdown in neurons, we found that CN06 incubation rescued the defects in dendritic growth and branching (Fig. 7*E*, *F*). These findings establish a molecular process in which KIDLIA knockdown induces an increase in membrane-bound N-cadherin and sequestration of cytosolic δ-catenin, leading to activation of the RhoA pathway and a subsequent alteration in actin dynamics, eventually causing suppression in neurite growth and branching (Fig. 7*G*).

## Discussion

Our study provides the first evidence showing that KIDLIA, the protein product of the recently identified XLID gene KIAA2022, plays an important role in neuron migration and morphogenesis. In mouse brain, knockdown of KIDLIA results in a redistribution of more neurons in the cortical plate at earlier time points and a subtle, but potentially consequential, mislocalization of neurons within their destined cortical layers. In addition, we find that early growth of the leading apical neurite is positively regulated in KIDLIA knockdown neurons; however, at a later time point (P14), loss of KIDLIA causes an inhibition in apical dendrite growth and a disruption of dendritic orientation. Given that IUE transfects only a small portion of neurons derived at a specific time and the majority of the neighboring neurons remain unaffected, the effects of KIDLIA are likely neuron autonomous, rather than being caused by external environmental factors.

How neuron migration is regulated by KIDLIA remains to be investigated. KIDLIA protein expression was mostly restricted to the cortical plate, with a distinct band of expression turning on in the subplate region just before the CP at E17 in mouse brain. IUE of KIDLIA shRNA led to more neurons migrating to upper cortical layers with a closer positioning of the soma to the pia. After migration, neurons reached their proper laminar layers; however, the aberrant positioning of the soma relative to the pia may indicate a disruption in the termination of the migration processor the final somal translocation after migration to layer II/III. However, we observed no change in the multipolar transition phase before entry into the CP. Future studies using *in utero* electroporation of KIDLIA shRNA at E12.5 to label layer V/VI neurons would be useful to investigate whether loss of KIDLIA could mislocalize deeper-layer neurons to upper cortical layers. Additionally, aberrant soma positioning could be due to premature cell cycle exit during proliferation, resulting in an earlier arrival of neurons to their destined cortical layers. The distinct expression pattern of KIDLIA within and just below the CP in embryonic brains suggests that KIDLIA may act as a checkpoint factor for CP entry; a loss of KIDLIA would therefore offer neurons free access to pass the IZ-CP border and enter into the CP.

We have observed similar changes in neurite growth in cultured neurons. KIDLIA knockdown led to a significant reduction in dendrite length and complexity. Using this *in vitro* system, we investigated the cellular mechanisms responsible for the disrupted dendritic development. We found that loss of KIDLIA induced an increase in total N-cadherin levels, with a substantial increase in the surfaced-localized fraction, which was accompanied by an elevated association of surface N-cadherin with δ-catenin. The increased δ-catenin/N-cadherin association led to depletion of the free cytosolic pool of δ-catenin, thereby increasing the activation of the downstream RhoA pathway. The RhoA pathway is a major mediator of actin dynamics and neurite outgrowth and complexity. Extensively branched processes can be induced when RhoA is inhibited via δ-catenin. Conversely, when RhoA is activated, dendrite length and the dendritic field are decreased (Li et al., 2000; Nakayama et al., 2000; Wong et al., 2000). The reduction in δ-catenin availability is responsible for the KIDLIA-dependent effect on dendrite morphogenesis, because overexpression of δ-catenin rescued neurite growth and dendritic branching as well as actin dynamics in neurons after KIDLIA knockdown. In line with our findings that δ-catenin is a key mediator for the neuronal effects of KIDLIA, δ-catenin was identified as a major genetic target in the autism population (Turner et al., 2015). Interestingly, of the autism genes positively correlated with δ-catenin, there is a significant enrichment in genes involved in dendrite morphogenesis, including PDLIM5, SHANK1, CDKL5, and DLG4 (Turner et al., 2015). In addition, loss of δ-catenin is implicated in impaired cognitive function and intellectual disabilities (Medina et al., 2000; Belcaro et al., 2015; Hofmeister et al., 2015), indicating a crucial role for δ-catenin in brain development and function.

Under the condition of KIDLIA knockdown, we found an increase in N-cadherin protein levels and an elevated association between N-cadherin and δ-catenin, but the underlying mechanism remains unclear. It is possible that a lack of KIDLIA expression in the nucleus leads to upregulation in N-cadherin gene transcription and subsequent protein translation. Regarding KIDLIA-induced changes in protein interaction, it has been shown that δ-catenin is subject to protein palmitoylation (Kang et al., 2008), a modification that causes enhanced association of δ-catenin with N-cadherin (Brigidi et al., 2014). Also, by binding to the juxtamembrane domain of cadherin, δ-catenin is known to stabilize and increase the amount of N-cadherin at the surface (Reynolds and Carnahan, 2004). It is thus conceivable that a loss of KIDLIA may cause δ-catenin palmitoylation, possibly via enhancing the expression of the palmitoyl-acyl transferase DHHC5 (Brigidi et al., 2014), leading to higher levels of association between δ-catenin and N-cadherin.

We found that the N-cadherin/δ-catenin effect is mediated by RhoA. The Rho GTPases regulate dendrite growth and branching via modulation of cytoskeleton components (Newey et al., 2005). The role for RhoA in dendritic branching has recently been established, as RhoA activation leads to a reduction in dendritic branching (Nakayama et al., 2000), whereas RhoA inhibition enhances branching (Neumann and Schweigreiter, 2002). Cytoplasmic δ-catenin has previously been shown to inhibit RhoA by keeping it in the inactive RhoA-GDP state, resulting in an increase in dendritic branching (Martinez et al., 2003). Constitutively active RhoA expression has been shown to reduce dendrite length and the volume of the dendritic field (Nakayama et al., 2000; Wong et al., 2000), whereas RhoA loss-of-function mutations have also been shown to cause abnormal dendritic arborization (Lee et al., 2000).

RT-PCR of mRNA prepared from prenatal and postnatal mouse brain shows a sevenfold increase in KIDLIA expression between E10.5 and E18.5, indicating a role for KIDLIA in neural development. KIDLIA mRNA expression reaches a maximum at P3 and is maintained at a low level into adulthood (Cantagrel et al., 2004; Ishikawa et al., 2012). Thus, KIDLIA expression peaks during key developmental time periods for neuronal growth and maturation. In both *in vivo* in mouse brain and cultured neurons, immunostainings reveal colocalization of KIDLIA with the nuclear marker Hoechst. KIDLIA is a large 170-kDa protein with monopartite and bipartite nuclear localization sequences. Given the specific nuclear localization, we suspect a major role for KIDLIA in gene regulation. It is possible that KIDLIA knockdown causes upregulation in N-cadherin gene transcription and thus an elevated amount of N-cadherin.

Attenuated growth and structural abnormalities in developing neurons lead to deficiencies in neuronal wiring and synapse formation during brain maturation. Aberrant neuron morphogenesis and synaptogenesis are associated with a number of brain disorders, including fragile-X mental retardation, Rett syndrome, Down syndrome, and CDKL5-related encephalopathy (Belmonte and Allen, 2004; Shepherd and Katz, 2011; Garner and Wetmore, 2012; Ricciardi et al., 2012). Given that KIDLIA is a novel gene with largely unknown functions, more studies are needed to elucidate the role of KIDLIA in multiple steps of neuron development including neurogenesis, morphogenesis, synapse maturation, synaptic plasticity, and behavior.

## Synthesis

The decision was a result of the Reviewing Editor Robert Kalb and the peer reviewers coming together and discussing their recommendations until a consensus was reached. A fact-based synthesis statement explaining their decision and outlining what is needed to prepare a revision is listed below. The following reviewer(s) agreed to reveal their identity: Marco Boccitto

Both reviewers were enthusiastic about the studies presented. In addition, both felt that data were being over interpreted (in specific experiments) or there was unclarity. The authors must re-write the ms. taking into account all of the editorial issues raised by both reviewers (in figure legends and the body of the ms.).

In addition, several addition experiments are required as outlined by reviewer #1:

The authors should validate the efficacy of their siRNA transfections. While the authors show a reduction of KIDLIA protein levels 6 days after shRNA infection, there is no validation of successful KIDLIA depletion 4 days after siRNA transfection. Even though the target sequence of the shRNA and siRNA is the same, validation of this depletion technique is an important control for the FRAP analysis.

6.Figure 7. Panel A should have a control blot in the IP panel for GST or Rhotekin. You can not conclude that there is more activated RhoA in the lysate without demonstrating that you are loading equivalent amounts of Rhotekin in the IP.

REgardingh rthe number of transduced cells the authors cite Ip et al and Takeuchi etal.

While both papers use percentage based approaches for their statistical analysis, they provide an accounting of the absolute number of cells that were compared between each group. I think this is important for addressing the concern previously raised by Reviewer #1.

Reviewer Comments:

Reviewer #1:

1.On page 16, the following sentence should be corrected:

"Indeed, after knockdown of KIDLIA we found a 35% increase in N-cadherin mRNA compared to virus of scrambled shRNA or untreated controls (Figure 5C)."

2.The authors should validate the efficacy of their siRNA transfections. While the authors show a reduction of KIDLIA protein levels 6 days after shRNA infection, there is no validation of successful KIDLIA depletion 4 days after siRNA transfection. Even though the target sequence of the shRNA and siRNA is the same, validation of this depletion technique is an important control for the FRAP analysis.

3.Figure 6. The legends for panels E and F incorrectly attribute the cells as being treated with shRNA.

4.Figure 7. The legend for panel D incorrectly attributes the cells as being treated with shRNA.

5.On page 18, "Indeed, FRAP revealed that in delta-catenin transfected neurons, actin dynamics were restored (Figure 6D) and the mobile and immobile fractions of actin returned to levels similar to controls (Figure 6E and 6F), indicating that the impaired actin dynamics likely resulted from a reduction of delta-catenin." There is no evidence for a reduction in delta-catenin. Should more accurately say a reduction in free cytoplasmic delta-catenin.

6.Figure 7. Panel A should have a control blot in the IP panel for GST or Rhotekin. You can not conclude that there is more activated RhoA in the lysate without demonstrating that you are loading equivalent amounts of Rhotekin in the IP.

7.As previously pointed out by Reviewer #1, there is currently insufficient evidence to attribute the changes in migration to increased migration speed. On page 20 the authors state that "In mouse brain, knockdown of KIDLIA results in facilitated neuron migration". More accurate/conservative language should be used here to describe the abnormalities in migration of KIDLIA depleted neurons.

8.Another point brought up previously by Reviewer #1 was the fact that the number of GFP positive cells in the scrambled shRNA IUE appears to be less than the KIDLIA shRNA IUE. While I agree with the authors that the percentage of cells in each layer is the relevant factor, I think it is important for the reader to have a sense of the total electroporated population in each group that was compared. The authors cite Ip et al. 2012, and Takeuchi and O'Leary 2006 to support their percentage based approach.

Ip et al., 2012

(d) Quantification of the distribution of GFP+ neurons. ***P > 0.001; Student's t-test. More than 1,000 GFP+ neurons from three to five brains were analyzed in each group

Takeuchi and O'Leary, 2006

The laminar (i.e., radial) distributions of GFP-labeled cells were quantified at late P0 from sections of electroporated brains with very similar transfection domains (five brains electroporated with the M1 RNAi vector and five with control vectors: one M6 RNAi and four E1 empty vectors). In the controls, the mean number of GFP-labeled cells is 370 {plus minus} 29 (total of 1851; range, 270-448); the M1 cases have a mean of 268 {plus minus} 30 labeled cells (total of 1342; range, 196-364).

While both papers use percentage based approaches for their statistical analysis, they provide an accounting of the absolute number of cells that were compared between each group. I think this is important for addressing the concern previously raised by Reviewer #1.

Reviewer #2:

ABSTRACT KIDLIA is said to be a syndrome, but then is being used in sentences of the abstract as if it were a protein.

The KIAA2022 protein has been called XPN in 6 publications, but a pubmed search for KIDLIA gives no results. What is the rationale for renaming this protein for this manuscript?

Most of the human genetics studies of patients with XPN mutations suffer from severe intellectual disability, along with symptoms that can be considered in the autistic spectrum. Since ASD in the context of severe intellectual disability and epilepsy can be very hard to compare to the majority of ASD instances, it should be clearly stated that this gene is p about a severe intellectual disability-epilepsy-ASD subvariant. The paper seems to imply in some place that the story is about autism per se.

"Interestingly, although KIDLIA

knockdown resulted in changes in the relative layer distribution of the migrating neurons, it did not affect their final laminar destination, with the majority of the electroporated neurons residing in layer II/III (Figure 2E)."

Since the timing of the electroporation is expected to label layer 2/3 neurons, and the migratory phenotype seems to be one of more rapid cell cycle exit or movement to the top of the cortical plate, that they end up in layer 2/3 like control is not surprising or particularly "interesting".

It would be interesting to know whether E12.5 electroporations result in mis-lamination of E12 born layer 5/6 neurons to layer 2/3.

For this journal the E12 IUEp is not necessary, but the fact that this would be a more definitive experiment should be mentioned in the discussion. In addition, the possibility that the phenotype could be due to premature cell cycle exit should also be discussed if not examined by, for example, E14 iuep then analysis of co-labeling for mitotic markers 24 hours later.

What is counted as an "N" in B and C of fig. 5. The legend says N=3 but the error is unusually small.

## References

[B1] Abu-Elneel K, Ochiishi T, Medina M, Remedi M, Gastaldi L, Caceres A,. (2008) A delta-catenin signaling pathway leading to dendritic protrusions. J Biol Chem 283:32781–32791. 10.1074/jbc.M804688200 18809680

[B2] Arikkath J, Israely I, Tao Y, Mei L, Liu X, Reichardt LF (2008) Erbin controls dendritic morphogenesis by regulating localization of delta-catenin. J Neurosci 28:7047–7056. 10.1523/JNEUROSCI.0451-08.2008 18614673 PMC2627506

[B3] Baio J (2014) Prevalence of autism spectrum disorder among children aged 8 years—autism and developmental disabilities monitoring network, 11 sites, United States, 2010. MMWR 63:1–21. 24670961

[B4] Bard L, Boscher C, Lambert M, Mège RM, Choquet D, Thoumine O (2008) A molecular clutch between the actin flow and N-cadherin adhesions drives growth cone migration. J Neurosci 28:5879–5890. 10.1523/JNEUROSCI.5331-07.2008 18524892 PMC6670336

[B5] Belcaro C, Dipresa S, Morini G, Pecile V, Skabar A, Fabretto A (2015) CTNND2 deletion and intellectual disability. Gene 565:146–149. 10.1016/j.gene.2015.03.054 25839933

[B6] Belichenko P, Wright E, Belichenko N, Masliah E, Li H, Mobley W,. (2009) Widespread changes in dendritic and axonal morphology in Mecp2-mutant mouse models of Rett syndrome: evidence for disruption of neuronal networks. J Comp Neur 514:240–258. 10.1002/cne.22009 19296534

[B7] Belmonte MK, Allen G (2004) Autism and abnormal development of brain connectivity. J Neurosci 24:9228–9231. 10.1523/JNEUROSCI.3340-04.2004 15496656 PMC6730085

[B8] Brigidi GS, Sun Y, Beccano-Kelly D, Pitman K, Mobasser M, Borgland SL, et al. (2014) Palmitoylation of delta-catenin by DHHC5 mediates activity-induced synapse plasticity. Nat Neurosci 17:522–532. 10.1038/nn.3657 24562000 PMC5025286

[B9] Cantagrel V, Haddad M-R, Ciofi P, Andrieu D, Lossi A-M, Maldergem L, et al. (2009) Spatiotemporal expression in mouse brain of Kiaa2022, a gene disrupted in two patients with severe mental retardation. Gene Express Patterns 9:423–429. 10.1016/j.gep.2009.06.001 19524067

[B10] Cantagrel V, Lossi AM, Boulanger S, Depetris D, Mattei MG, Gecz J, et al. (2004) Disruption of a new X linked gene highly expressed in brain in a family with two mentally retarded males. J Med Genet 41:736–742. 10.1136/jmg.2004.021626 15466006 PMC1735597

[B11] Charzewska A, Rzońca S, Janeczko M, Nawara M, Smyk M, Bal J, et al. (2014) A duplication of the whole KIAA2022 gene validates the gene role in the pathogenesis of intellectual disability and autism. Clin Genet 88:297–299. 10.1111/cge.12528 25394356

[B12] de Anda F, Rosario A, Durak O, Tran T, Gräff J, Meletis K, et al. (2012) Autism spectrum disorder susceptibility gene TAOK2 affects basal dendrite formation in the neocortex. Nat Neurosci 15:1022–1031. 10.1038/nn.3141 22683681 PMC4017029

[B13] DiCicco-Bloom E, Lord C, Zwaigenbaum L, Courchesne E, Dager S, Schmitz C, et al. (2006) The developmental neurobiology of autism spectrum disorder. J Neurosci 26:6897–6906. 10.1523/JNEUROSCI.1712-06.2006 16807320 PMC6673916

[B14] Elia L, Yamamoto M, Zang K, Reichardt L (2006) p120 catenin regulates dendritic spine and synapse development through Rho-family GTPases and cadherins. Neuron 51:43–56. 10.1016/j.neuron.2006.05.018 16815331 PMC2587166

[B15] Gal JS, Morozov YM, Ayoub AE, Chatterjee M, Rakic P, Haydar TF (2006) Molecular and morphological heterogeneity of neural precursors in the mouse neocortical proliferative zones. J Neurosci 26:1045–1056. 10.1523/JNEUROSCI.4499-05.2006 16421324 PMC3249619

[B16] Garner CC, Wetmore DZ (2012) Synaptic pathology of Down syndrome. Adv Exp Med Biol 970:451–468. 10.1007/978-3-7091-0932-8_20 22351068

[B17] Hofmeister W, Nilsson D, Topa A, Anderlid BM, Darki F, Matsson H, et al. (2015) CTNND2—a candidate gene for reading problems and mild intellectual disability. J Med Genet 52:111–122. 10.1136/jmedgenet-2014-102757 25473103

[B18] Ishikawa T, Miyata S, Koyama Y, Yoshikawa K, Hattori T, Kumamoto N, et al. (2012) Transient expression of Xpn, an XLMR protein related to neurite extension, during brain development and participation in neurite outgrowth. Neurosci 214:181–191. 10.1016/j.neuroscience.2012.04.030 22531377

[B19] Ishizaki T, Uehata M, Tamechika I, Keel J, Nonomura K, Maekawa M, et al. (2000) Pharmacological properties of Y-27632, a specific inhibitor of rho-associated kinases. Mol Pharmacol 57:976–983. 10779382

[B20] Kang R, Wan J, Arstikaitis P, Takahashi H, Huang K, Bailey AO, et al. (2008) Neural palmitoyl-proteomics reveals dynamic synaptic palmitoylation. Nature 456:904–909. 10.1038/nature07605 19092927 PMC2610860

[B21] Kim H, Oh M, Lu Q, Kim K (2008) E-Cadherin negatively modulates delta-catenin-induced morphological changes and RhoA activity reduction by competing with p190RhoGEF for delta-catenin. Biochem Biophys Res Commun 377:636–641. 10.1016/j.bbrc.2008.10.030 18930028 PMC2614342

[B22] Kozma R, Sarner S, Ahmed S, Lim L (1997) Rho family GTPases and neuronal growth cone remodelling: relationship between increased complexity induced by Cdc42Hs, Rac1, and acetylcholine and collapse induced by RhoA and lysophosphatidic acid. Mol Cell Biol 17:1201–1211. 9032247 10.1128/mcb.17.3.1201 PMC231845

[B23] Kuroda Y, Ohashi I, Naruto T, Ida K, Enomoto Y, Saito T, et al. (2015) Delineation of the KIAA2022 mutation phenotype: two patients with X linked intellectual disability and distinctive features. Am J Med Genet a 167:1349–1353. 10.1002/ajmg.a.37002 25900396

[B24] Kwon C-H, Luikart B, Powell C, Zhou J, Matheny S, Zhang W, et al. (2006) Pten regulates neuronal arborization and social interaction in mice. Neuron 50:377–388. 10.1016/j.neuron.2006.03.023 16675393 PMC3902853

[B25] Lee T, Winter C, Marticke SS, Lee A, Luo L (2000) Essential roles of Drosophila RhoA in the regulation of neuroblast proliferation and dendritic but not axonal morphogenesis. Neuron 25:307–316. 10719887 10.1016/s0896-6273(00)80896-x

[B26] Leeuwen FN, Kain HE, Kammen RA, Michiels F, Kranenburg OW, Collard JG (1997) The guanine nucleotide exchange factor Tiam1 affects neuronal morphology; opposing roles for the small GTPases Rac and Rho. J Cell Biol 139:797–807. 10.1083/jcb.139.3.797 9348295 PMC2141700

[B27] Li Z, Aelst VL, Cline HT (2000) Rho GTPases regulate distinct aspects of dendritic arbor growth in Xenopus central neurons in vivo. Nat Neurosci 3:217–225. 10700252 10.1038/72920

[B28] Lord C, Rutter M, Goode S, Heemsbergen J, Jordan H, Mawhood L, et al. (1989) Autism diagnostic observation schedule: a standardized observation of communicative and social behavior. J Autism Dev Disord 19:185–212. 2745388 10.1007/BF02211841

[B29] Lu Q, Paredes M, Medina M, Zhou J (1999) δ-Catenin, an adhesive junction–associated protein which promotes cell scattering. J Cell Biol 144:519–532. 9971746 10.1083/jcb.144.3.519 PMC2132907

[B30] Magome T, Hattori T, Taniguchi M, Ishikawa T, Miyata S, Yamada K, et al. (2013) XLMR protein related to neurite extension (Xpn/KIAA2022) regulates cell-cell and cell-matrix adhesion and migration. Neurochem Int 63:561–569. 10.1016/j.neuint.2013.09.011 24071057

[B31] Martinez M, Ochiishi T, Majewski M, Kosik KS (2003) Dual regulation of neuronal morphogenesis by a delta-catenin–cortactin complex and Rho. J Cell Biol 162:99–111. 10.1083/jcb.200211025 12835311 PMC2172717

[B32] Meberg PJ, Bamburg JR (2000) Increase in neurite outgrowth mediated by overexpression of actin depolymerizing factor. J Neurosci 20:2459–2469. 10729326 10.1523/JNEUROSCI.20-07-02459.2000 PMC6772241

[B33] Medina M, Marinescu RC, Overhauser J, Kosik KS (2000) Hemizygosity of δ-catenin (CTNND2) is associated with severe mental retardation in cri-du-chat syndrome. Genomics 63:157–164. 10.1006/geno.1999.6090 10673328

[B34] Nakayama AY, Harms MB, Luo L (2000) Small GTPases Rac and Rho in the maintenance of dendritic spines and branches in hippocampal pyramidal neurons. J Neurosci 20:5329–5338. 10884317 10.1523/JNEUROSCI.20-14-05329.2000 PMC6772334

[B35] Neumann H, Schweigreiter R (2002) Tumor necrosis factor inhibits neurite outgrowth and branching of hippocampal neurons by a rho-dependent mechanism. J Neurosci 22:854–862. 11826115 10.1523/JNEUROSCI.22-03-00854.2002 PMC6758473

[B36] Newey SE, Velamoor V, Govek EE (2005) Rho GTPases, dendritic structure, and mental retardation. Dev Neurobiol 64:58–74. 10.1002/neu.20153 15884002

[B37] Nguyen D, Disteche C (2006) High expression of the mammalian X chromosome in brain. Brain Res 1126:46–49. 10.1016/j.brainres.2006.08.053 16978591

[B38] Nicholson-Dykstra S, Higgs HN, Harris ES (2005) Actin dynamics: growth from dendritic branches. Curr Biol 15:R346–3R357. 10.1016/j.cub.2005.04.029 15886095

[B39] Ouyang Q, Lizarraga SB, Schmidt M, Yang U, Gong J, Ellisor D, et al. (2013) Christianson syndrome protein NHE6 modulates TrkB endosomal signaling required for neuronal circuit development. Neuron 80:97–112. 10.1016/j.neuron.2013.07.043 24035762 PMC3830955

[B40] Peça J, Feliciano C, Ting JT, Wang W, Wells MF, Venkatraman TN, et al. (2011) Shank3 mutant mice display autistic-like behaviours and striatal dysfunction. Nature 472:437–442. 10.1038/nature09965 21423165 PMC3090611

[B41] Reynolds AB, Carnahan RH (2004) Regulation of cadherin stability and turnover by p120ctn: implications in disease and cancer. Semin Cell Dev Biol 15:657–663. 10.1016/j.semcdb.2004.09.003 15561585

[B42] Ricciardi S, Ungaro F, Hambrock M, Rademacher N, Stefanelli G, Brambilla D, et al. (2012) CDKL5 ensures excitatory synapse stability by reinforcing NGL-1-PSD95 interaction in the postsynaptic compartment and is impaired in patient iPSC-derived neurons. Nat Cell Biol 14:911–923. 10.1038/ncb2566 22922712 PMC6485419

[B43] Shepherd GM, Katz DM (2011) Synaptic microcircuit dysfunction in genetic models of neurodevelopmental disorders: focus on Mecp2 and Met. Curr Opin Neurobiol 21:827–833. 10.1016/j.conb.2011.06.006 21733672 PMC3199024

[B44] Skuse D (2005) X-linked genes and mental functioning. Hum Mol Genet 14 Spec No 1:32. 10.1093/hmg/ddi112 15809269

[B45] Tan Z-JJ, Peng Y, Song H-LL, Zheng J-JJ, Yu X (2010) N-cadherin-dependent neuron-neuron interaction is required for the maintenance of activity-induced dendrite growth. Proc Natl Acad Sci U S A 107:9873–9878. 10.1073/pnas.1003480107 20457910 PMC2906874

[B46] Turner TN, Sharma K, Oh EC, Liu YP, Collins RL, Sosa MX, et al. (2015) Loss of delta-catenin function in severe autism. Nature 520:51–56. 10.1038/nature14186 25807484 PMC4383723

[B47] Van Maldergem L, Hou Q, Kalscheuer V, Rio M, Doco-Fenzy M, Medeira A, et al. (2013) Loss of function of KIAA2022 causes mild to severe intellectual disability with an autism spectrum disorder and impairs neurite outgrowth. Hum Mol Genet 22:3306–3314. 10.1093/hmg/ddt187 23615299 PMC3723314

[B48] Weston MC, Chen H, Swann JW (2014) Loss of mTOR repressors Tsc1 or Pten has divergent effects on excitatory and inhibitory synaptic transmission in single hippocampal neuron cultures. Front Mol Neurosci 7:1 10.3389/fnmol.2014.00001 24574959 PMC3922082

[B49] Wong WT, Faulkner-Jones BE, Sanes JR (2000) Rapid dendritic remodeling in the developing retina: dependence on neurotransmission and reciprocal regulation by Rac and Rho. J Neurosci 20:5024–5036. 10864960 10.1523/JNEUROSCI.20-13-05024.2000 PMC6772263

[B50] Yu X, Malenka RC (2003) Beta-catenin is critical for dendritic morphogenesis. Nat Neurosci 6:1169–1177. 10.1038/nn1132 14528308

[B51] Zikopoulos B, Barbas H (2010) Changes in prefrontal axons may disrupt the network in autism. J Neurosci 30:14595–14609. 10.1523/JNEUROSCI.2257-10.2010 21048117 PMC3073590

